# Hepatocyte-Specific ApoJ Knockout Improves Metabolic Profiles in the Liver of Diabetic Mice

**DOI:** 10.3390/metabo15120761

**Published:** 2025-11-25

**Authors:** Sin-Tian Wang, Xing-Min Li, Jiayi Pi, Yu-Ting Hsu, Li-Chi Chi, Hung-Yu Sun

**Affiliations:** 1 Department of Medical Laboratory Science and Biotechnology, College of Medicine, National Cheng Kung University, Tainan 701401, Taiwan; 11003058@gs.ncku.edu.tw (S.-T.W.); i34129516@gs.ncku.edu.tw (X.-M.L.); 2 College of Biology, Hunan University, Changsha 410082, China; jiayi-pi@hnu.edu.cn; 3 Department of Physiology, College of Medicine, National Cheng Kung University, Tainan 701401, Taiwan; s36134126@gs.ncku.edu.tw (Y.-T.H.); lcchi@gs.ncku.edu.tw (L.-C.C.); 4 Institute of Basic Medicine, College of Medicine, National Cheng Kung University, Tainan 701401, Taiwan

**Keywords:** Apolipoprotein J, type-II diabetes, hepatic metabolism, tissue-specific knockout

## Abstract

**Background/Objectives:** Type 2 diabetes mellitus (T2DM) is a major metabolic disorder and is frequently accompanied by liver steatosis. Apolipoprotein J (ApoJ) is a glucose-regulated molecular chaperone that has been implicated in hepatic lipid deposition under nutrient overload. This study aimed to investigate the role of hepatocyte-specific ApoJ deletion in hepatic metabolism under diabetic conditions. **Methods:** A T2DM mouse model with hepatocyte-specific ApoJ knockout (HKO) was established through a high-fat diet combined with streptozotocin injection. Hepatic metabolic profiles were analyzed using untargeted metabolomics with UHPLC–MS/MS. Differential metabolites were subjected to KEGG pathway and Sankey diagram analyses to identify biologically relevant pathways. **Results:** In total, 140 metabolites showed significant differential abundance in HKO mouse liver, primarily encompassing organic acids and derivatives as well as lipids and lipid-like molecules. KEGG analysis revealed that ApoJ deletion enhanced pathways related to vitamin digestion and absorption, thiamine metabolism, amino acid biosynthesis, lysine degradation, and 2-oxocarboxylic acid metabolism. In contrast, pathways associated with galactose metabolism, cysteine and methionine metabolism, purine metabolism, and the pentose phosphate pathway were suppressed. Sankey diagram analysis further demonstrated that ApoJ deletion markedly reshapes hepatic metabolic networks in T2DM. **Conclusions:** Given the central role of hepatic dysmetabolism in the pathogenesis of diabetes and its complications, targeting ApoJ may represent a promising therapeutic approach for restoring hepatic metabolic homeostasis and preventing diabetes-associated steatosis.

## 1. Introduction

Diabetes mellitus affects over 830 million individuals worldwide and ranks as the eighth leading cause of mortality globally. More than 90% of these cases are classified as type 2 diabetes mellitus (T2DM), a condition frequently accompanied by features of metabolic dysfunction syndrome (MDS), including overweight or obesity, metabolic dysfunction-associated steatotic liver disease (MASLD), and dyslipidemia. A global epidemiological study further reported that approximately 28.3% of individuals with T2DM are diagnosed with MASLD, with regional prevalence ranging from 24.1% to 47.1% across different continents. This strong association underscores that T2DM and MASLD are interrelated manifestations of the same metabolic spectrum [1].

Apolipoprotein J (ApoJ), also known as Clusterin, is a glucose-regulated molecular chaperone [2]. ApoJ is ubiquitously expressed across human tissues, with the liver serving as the principal site of secretion [3]. Under normal physiological conditions, ApoJ functions as an accessory protein of lipoproteins and contributes to lipid transport [4]. Beyond this canonical role, ApoJ has been implicated in viral particle formation in hepatitis C virus-infected hepatocytes [4]. Our group has recently demonstrated that ApoJ acts as a pathogenic chaperone in pathogen- and nutrient-induced hepatic lipid accumulation [5,6], suggesting that it may play a broader role in maintaining metabolic homeostasis in the liver. Therefore, hepatic ApoJ represents a potential therapeutic target for correcting metabolic imbalances associated with metabolic disorders. Supporting this notion, an ApoJ-antagonizing peptide is shown to ameliorate hepatic lipid accumulation by promoting the proteasomal degradation of mTOR and enhancing autophagy–lysosomal activity [6]. Furthermore, an antisense oligonucleotide-targeting ApoJ (OGX-011) has been reported to regulate apoptotic homeostasis in cancer cells by preventing cytochrome c release into the cytoplasm through inhibition of the Ku70–BAX interaction and modulation of histone deacetylase inhibitor activity [2].

Given the central role of hepatic dysregulation in diabetes progression and its complications, we therefore hypothesized that targeting ApoJ may reshape hepatic homeostasis and prevent the development of T2DM. However, a knowledge gap remains regarding the influence of ApoJ on hepatic metabolism under diabetic conditions. In the present study, we aimed to examine the impact of hepatocyte-specific ApoJ deletion (HKO) on hepatic metabolism in a T2DM mouse model. Using untargeted metabolomics approaches, we identified widespread alterations in liver metabolites and demonstrated that ApoJ deletion significantly reshapes hepatic metabolic networks under diabetic conditions. Our findings suggest that ApoJ may represent a promising therapeutic target for preventing the development of MDS-induced T2DM.

## 2. Materials and Methods

### 2.1. Animals and Experimental Procedure

Hepatocyte-specific ApoJ knockout (HKO) mice were generated on a C57BL/6 background using the CRISPR/Cas9 system, as previously described [6]. Briefly, HKO mice (Clu-Flox/Alb-Cre-Tgc, cat no, NM-XA-252759) were obtained from the Shanghai Model Organisms Center, Inc. (Shanghai, China). The Clu-^flox/flox^ mice (C57BL/6Smoc-*Clu^em1(flox)Smoc^,* cat no, NM-CKO-210035) were generated by inserting the loxP fragment into the introns between exon 3–4 and exon 5–6 using the CRISPR/Cas9 system. The HKO mice were produced by crossing Clu^flox/flox^ mice with Alb-Cre transgenic (Alb-Cre) mice.

A T2DM model was established by administering a D12492 high-fat diet (HFD) for four weeks, followed by a single intraperitoneal injection of streptozotocin (STZ, 100 mg/kg). Successful induction of diabetes was confirmed by elevating fasting blood glucose levels two weeks after the STZ injection. Both Fox and HKO mice exhibited significant hyperglycemia, indicating successful establishment of the diabetic phenotype. The HFD was subsequently maintained for an additional 16 weeks to ensure persistence of the diabetic phenotype.

In each experimental group, four male mice (Flox or HKO) were used to examine metabolic changes in the livers of mice with T2DM induced by HFD/STZ. All experimental mice survived after the 16-week treatment. After the 16-week treatment, all mice were anesthetized with isoflurane and sacrificed by cervical dislocation. The liver tissues were immediately excised, rinsed with cold saline, and stored at −80 °C until further analysis.

All animals were housed under standardized conditions (22 °C; 12-h light/dark cycle; 50–60% relative humidity). All experimental procedures were conducted in strict accordance with the ARRIVE guidelines.

### 2.2. UHPLC-MS/MS Analysis

Metabolomic profiling of liver tissues was performed by Shanghai Bioprofile Technology Co., Ltd. (Shanghai, China). Liver tissue from Flox or HKO mice, matched for weight, was homogenized in normal saline using a grinding mill, and hepatic lysates were collected after centrifugation. Hepatic lysates were lyophilized, pulverized, and metabolites were extracted with methanol–acetonitrile–water (2:2:1, *v*/*v*/*v*) under ultrasonic agitation, followed by vacuum concentration. Metabolites separation was carried out on a UHPLC system (Shimadzu Nexera X2 LC-30AD, Shimadzu, Japan) coupled with a Q-Exactive Plus Orbitrap MS (Thermo Scientific, Fremont, CA, USA) using an ACQUITY UPLC^®^ HSS T3 column (2.1 × 100 mm, 1.8 μm) (Waters, Milford, MA, USA). The mobile phase consisted of 0.1% formic acid in water and acetonitrile, with a flow rate of 0.3 mL/min. Data were acquired in both positive and negative ionization modes (*m*/*z* 70–1050) with full MS resolution of 70,000 and MS/MS resolution of 17,500. Source parameters included spray voltages of 3.8 kV (positive) and 3.2 kV (negative), capillary temperature of 320 °C, and probe heater temperature of 350 °C.

The quality control (QC) samples were prepared by pooling equal aliquots from all individual samples to represent the overall study composition. These pooled QC samples were designed to represent the overall composition of the study cohort. QC samples were injected at regular intervals throughout the HPLC–MS/MS run to monitor instrument stability and potential signal drift.

Raw MS data from QC, Flox, and HKO samples were processed and peak-aligned using MS-DIAL software (ver.4.9.221218). Data from positive and negative ionization modes were processed separately to ensure mode-specific feature alignment and identification. Adducts were automatically identified and consolidated during feature annotation in MS-DIAL and quantification of small molecules via mass spectral deconvolution.

Features detected in fewer than 50% of all samples were excluded from further analysis. Metabolite identification was performed by matching accurate mass (<10 ppm) and MS/MS fragmentation patterns (<0.02 Da) against a proprietary database (Shanghai Bioprofile Technology Co., Ltd., Shanghai, China), which integrates multiple sources including MassBank, public databases, the Human Metabolome Database (HMDB), and an in-house metabolite standard library (Supplementary Tables S1 and S2). Missing values were imputed using the minimum observed value for each metabolite. Outliers were retained if they met QC-based reproducibility criteria. QC-based normalization was then applied to correct for batch effects and signal drift prior to statistical analysis. Analytical repeatability and system stability were assessed using principal component analysis (PCA) (Supplementary Figure S1A,B).

### 2.3. KEGG Enrichment Analysis

KEGG pathway analysis was conducted on the differential metabolite data using the KEGG database http://www.kegg.jp (accessed on 16 November 2022) to identify biologically relevant pathways. Pathway enrichment was assessed using Fisher’s exact test, with multiple testing correction applied using the false discovery rate (FDR) method. KEGG pathways with *p* < 0.05 were considered statistically significant.

### 2.4. Multivariate Statistical Analysis

Multivariate statistical analyses were conducted using R packages (version 4.0.3). Partial least squares discriminant analysis (PLS-DA) was applied to evaluate the metabolic variation and to discriminate between experimental groups. Variable importance in projection (VIP) scores derived from orthogonal partial least squares discrimination (OPLS-DA) models were used to assess metabolite contributions to group separation, with VIP > 1.0 indicating influential variables.

Complementary univariate analyses were performed on normalized data, employing two-tailed Student’s *t*-tests for pairwise comparisons and one-way ANOVA for multi-group comparisons. Metabolites with both VIP > 1.0 and *p* < 0.05 were defined as statistically significant. Fold changes were calculated as the log-transformed ratio of mean signal intensities between groups, and the differential metabolites were further subjected to hierarchical cluster using the ropls(R) software (version 1.28.2).

## 3. Results

### 3.1. Hepatocyte-Specific ApoJ Knockout Alters Hepatic Metabolism in Diabetic Mice

**Figure 1:**
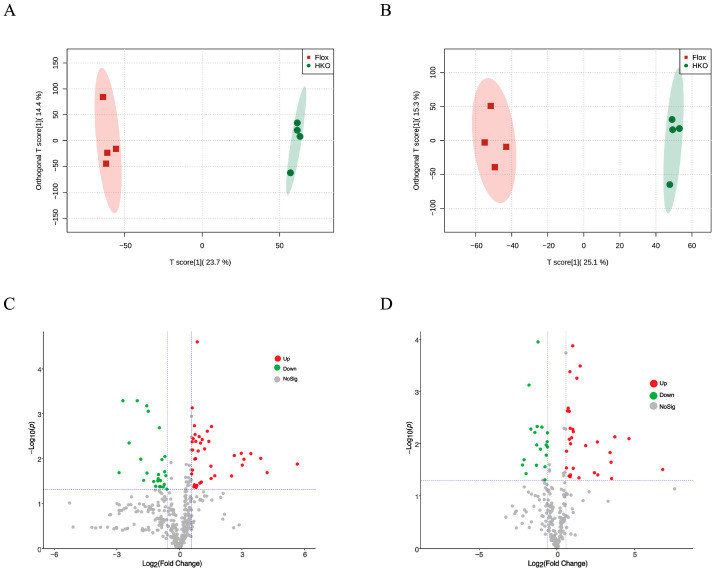
Metabolomic analysis of liver lysate from mice with STZ-induced diabetes. The OPLS-DA score plot of positive metabolites (**A**) and negative metabolites (**B**) in diabetic Flox and hepatocyte-specific ApoJ knockout (HKO) mice. The volcano plot analysis of positive (**C**) and negative (**D**) metabolites. The differentially increased metabolites were identified using the criteria Log2(FC) > 0.667 and *p*-value < 0.05, shown in red; differentially decreased metabolites were identified using the criteria Log2(FC) < −0.667 and *p*-value < 0.05, shown in green.

**Figure 2:**
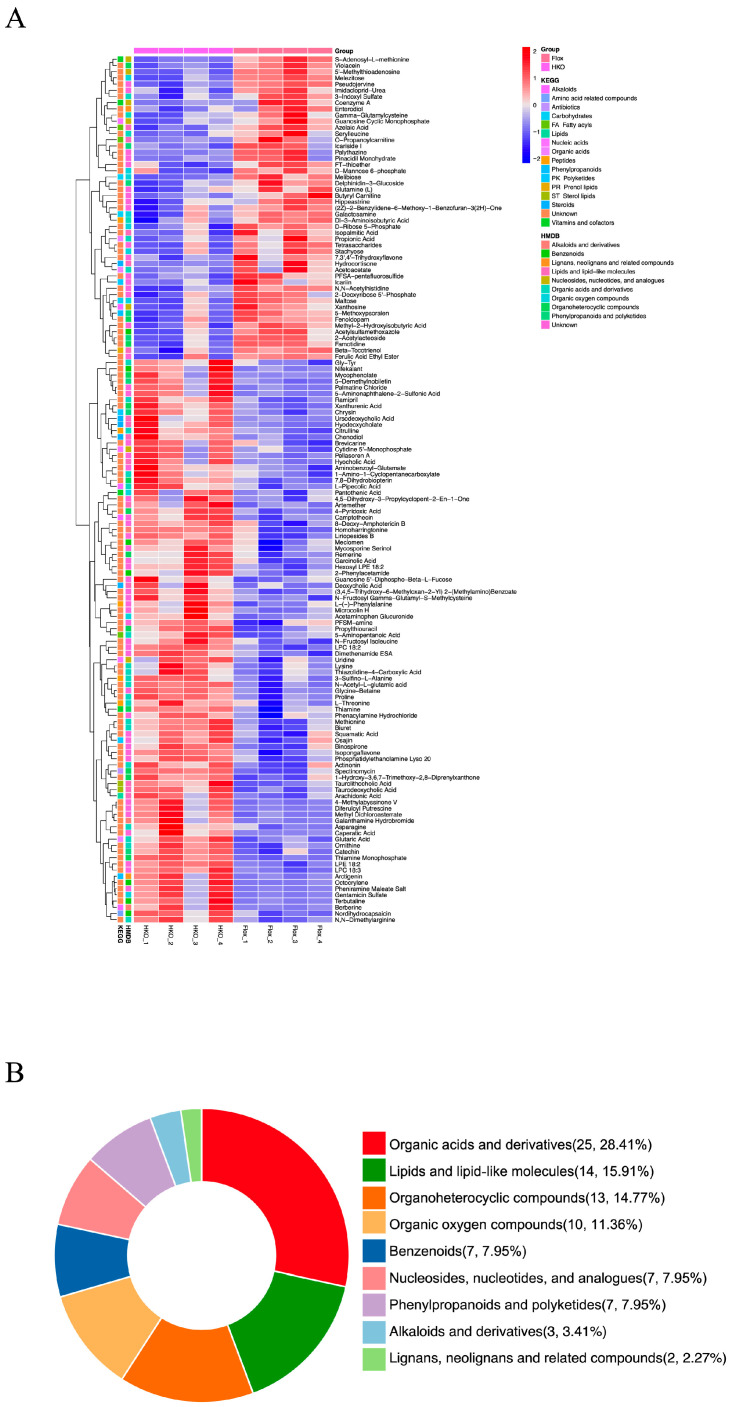
Differential metabolomic profiling in the liver of diabetic HKO mice. (**A**) Hierarchical clustering analysis of differentially abundant metabolites identified in Figure 1 via KEGG and Human Metabolome Database (HMDB). (**B**) Classification of differential abundance metabolites via HMDB.

Previously, we demonstrated that HKO mice exhibit improved glycemic and lipid profiles compared to Flox mice in the MASLD model [6]. To further investigate the role of ApoJ in hepatic metabolism, metabolomic analysis was performed on liver lysates from diabetic mice, comparing flox and HKO mice. The OPLS-DA score plots for both positive and negative metabolites showed a clear separation between the flox and HKO mice, indicating distinct metabolic profiles (Figure 1A,B). A total of 627 metabolites were identified in the HMDB, including 382 metabolites with positive loading values and 245 metabolites with negative loading values. Among these, 140 metabolites were found to be differentially abundant in the liver of diabetic HKO mice (Supplementary Materials). Hierarchical clustering based on KEGG and HMDB annotations revealed two distinct clusters (Figure 2A). These metabolites were classified into nine categories, with organic acids and derivatives (28.41%) and lipids and lipid-like molecules (15.91%) being the most prominent (Figure 2B).

### 3.2. Functional Assessment of Differentially Abundant Positive Metabolites

**Figure 3:**
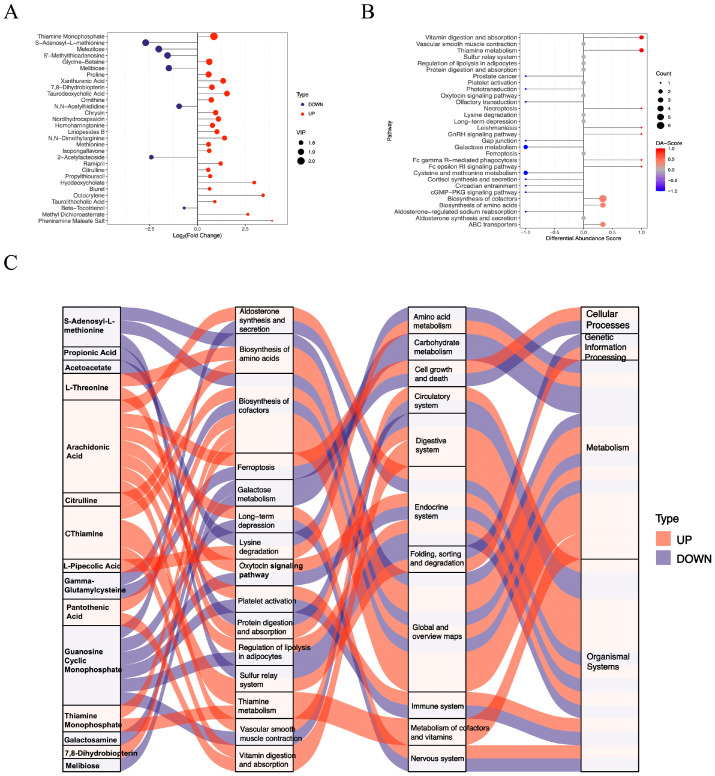
Functional analysis of differentially abundant positive-ion metabolites. (**A**) Lollipop plot showing the top 30 significantly altered metabolites. (**B**) Differential abundance (DA) scores highlighting upregulated and downregulated KEGG pathway. (**C**) Sankey diagram illustrating the mapping of upregulated (red lines) and downregulated (blue lines) metabolites to the enriched KEGG pathways.

In the livers of the diabetic HKO mice, 55 positively charged metabolites were significantly increased, whereas 27 were significantly decreased (Figure 1C). The top 30 significantly altered metabolites were ranked according to the VIP index (Figure 3A). Hepatic levels of thiamine monophosphate, glycine-betaine, proline, and ornithine were elevated, while S-adenosyl-L-methionine, melezitose, and 5′-methylthioadenosine were reduced. Differential abundance (DA) scores highlighted the impact on metabolic pathways (Figure 3B). The HKO condition enhanced pathways involved in vitamin digestion and absorption, thiamine metabolism, and the biosynthesis of amino acids and cofactors. In contrast, pathways related to galactose metabolism and cysteine and methionine metabolism were inhibited. A Sankey diagram visualized the flow of these metabolites through enriched KEGG pathways, underscoring the regulatory role of ApoJ in hepatic metabolic remodeling (Figure 3C).

### 3.3. Functional Assessment of Differentially Abundant Negative Metabolites

**Figure 4:**
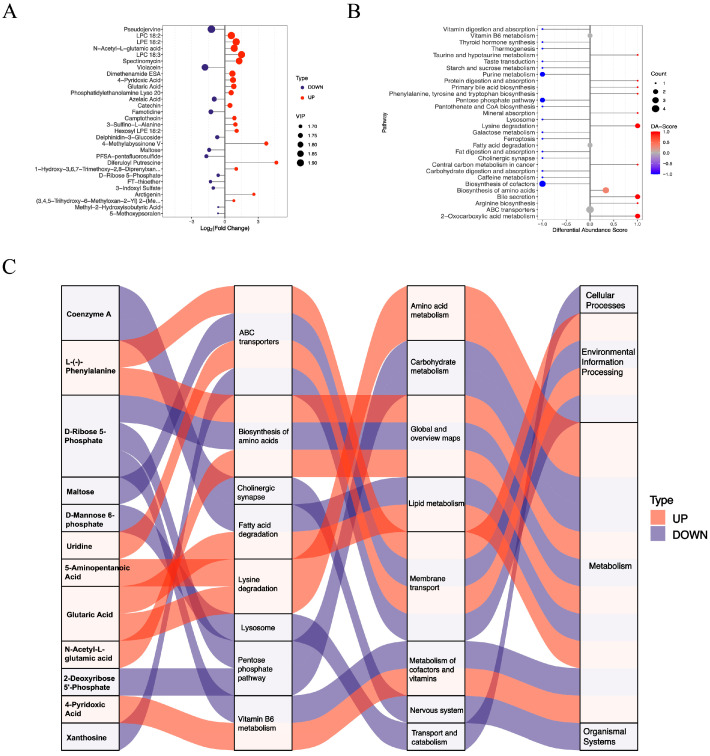
Functional analysis of differentially abundant negative-mode metabolites. (**A**) The top 30 differentially abundant metabolites detected in negative ion mode are displayed. (**B**) DA scores highlight significantly upregulated and downregulated KEGG pathways. (**C**) A Sankey diagram illustrating the mapping of upregulated (red lines) and downregulated (blue lines) metabolites to their corresponding enriched KEGG pathways, showing the flow of metabolic alterations.

Significant changes were also observed in 58 negatively charged metabolites in the livers of the diabetic HKO mice. Among these, 36 metabolites were increased and 22 were decreased abundance (Figure 1D). The top 30 significantly altered metabolites were identified based on VIP score (Figure 4A). Metabolites with increased abundance included lysophosphatidylcholines (LPC 18:2 and LPC 18:3), N-acetyl-L-glutamic acid, glutaric acid, and 4-pyridoxic acid. In contrast, violacein, maltose, D-ribose 5-phosphate, 3-indoxyl sulfate, and famotidine were reduced. KEGG pathway analysis revealed several enriched metabolic pathways (Figure 4B). Enhanced pathways included lysine degradation, biosynthesis of amino acids, and 2-oxocarboxylic acid metabolism, whereas purine metabolism, the pentose phosphate pathway, and the biosynthesis of cofactors were down regulated. A Sankey diagram illustrated the flow of these metabolites across enriched KEGG pathways, further emphasizing the critical role of ApoJ in hepatic metabolic regulation (Figure 4C).

## 4. Discussion

**Figure 5:**
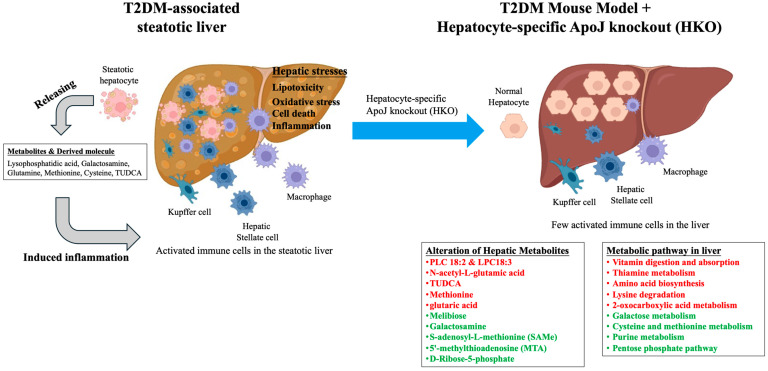
The hepatocyte-specific ApoJ knockout alters hepatic metabolites and reshapes metabolite-related pathways in the T2DM-associated steatotic liver. In T2DM, insulin resistance promotes hepatic lipid accumulation, leading to steatosis. The excessive production of metabolites such as lysophosphatidic acid [7], galactosamine [8], glutamine [9], methionine [10], cysteine [11], tauroursodeoxycholic acid (TUDCA) [12], along with their derivatives, disrupts hepatic metabolic balance within steatotic hepatocytes. These metabolites influence both parenchymal and non-parenchymal liver cells, amplifying hepatic stress responses including lipotoxicity, oxidative stress, cell death, and inflammation. In contrast, HKO appears to restore metabolic equilibrium and attenuate these stress responses. Therefore, HKO may contribute to the re-establishment of hepatic inflammatory homeostasis by reshaping dysregulated metabolic pathways. Upregulated metabolites and pathways are indicated in red, while downregulated metabolites and pathways are indicated in green.

Insulin resistance is fundamental metabolic disturbance in T2DM that promotes excessive hepatic lipid accumulation and the development of steatosis. The resulting dysregulation of lipid metabolism alters the hepatic lipid landscape and drives the aberrant production of various metabolic intermediates and signaling molecules. Among these, lysophosphatidic acid [7], galactosamine [8], glutamine [9], methionine [10], cysteine [11], tauroursodeoxycholic acid (TUDCA) [12], and their derivative metabolites have been identified as key contributors to hepatic metabolic stress. The overproduction and accumulation of these metabolites within steatotic hepatocytes can profoundly influence both hepatocyte and non-parenchymal cells, including Kupffer cells, hepatic stellate cells, and endothelial cells. This metabolic imbalance exacerbates hepatic insults through the induction of lipotoxicity, oxidative stress, endoplasmic reticulum stress, and subsequent inflammatory cascades, collectively aggravating liver injury and promoting disease progression. Conversely, hepatic ApoJ deletion appears to restore metabolic balance by modulating the hepatic metabolome and alleviating cellular stress responses. By normalizing key metabolic pathways and reducing the accumulation of toxic intermediates, HKO mitigates lipotoxic and inflammatory signaling within the hepatic microenvironment. Consequently, HKO may help re-establish hepatic homeostasis through the reshaping of dysregulated metabolic networks and the restoration of intercellular communication between hepatocytes and non-parenchymal cell populations (Figure 5).

Lysophosphatidylcholines (LPCs), particularly LPC 18:2 and LPC 18:3, have been implicated in both type 1 and type 2 diabetes mellitus. Low plasma levels of LPC 18:2 are associated with an increased risk of T2DM, insulin resistance and impaired glucose tolerance [13], whereas higher LPC concentrations have been linked to reduced diabetes risk [14]. Similarly, lysophosphatidylethanolamine (LPE) 18:2, a specific LPE lipid, is inversely associated with diabetes and cardiovascular disease [15]. In the present study, HKO mice exhibited elevated hepatic levels of both LPC and LPE, suggesting that ApoJ influences phospholipid metabolism remodeling, possibly through regulation of phospholipid metabolism or lipoprotein assembly. The observed increase in LPC and LPE in HKO livers implies that loss of ApoJ function could enhance the availability of these protective lipids, thereby improving insulin sensitivity and metabolic homeostasis in T2DM. This mechanistic link highlights ApoJ as a potential therapeutic target for correcting lipid dysregulation in diabetes.

Insulin resistance drives the pathogenesis of metabolic-associated steatotic liver disease (MASLD) by promoting de novo lipogenesis [16]. The resulting lipid overload triggers oxidative stress, which damages hepatocytes and dysregulates insulin signaling [17], and triggers chronic inflammation through activation of resident macrophages and recruitment of immune cells [18]. Together, insulin resistance, oxidative stress, and inflammation form an interdependent network that drives the progression of MASLD from steatosis to steatohepatitis and fibrosis.

Dysregulation of the identified pathways, including galactose metabolism, cysteine and methionine metabolism, purine metabolism, and the pentose phosphate pathway (PPP), are well-established contributors to metabolic disorders such as type 2 diabetes and metabolic syndrome. Elevated plasma galactose and alterations in galactose metabolism have been reported in individuals with prediabetes [19]. Disruption in cysteine and methionine metabolism, including increased S-adenosylmethionine (SAMe) and S-adenosylhomocysteine (SAH), impair hepatic methylation reactions and insulin signaling [20,21,22]. In our HKO mice, modulation of SAMe and SAH suggests that ApoJ may influence amino acid-related methylation pathways, contributing to improved insulin sensitivity. Likewise, metabolites such as N-acetyl-L-glutamic acid [23,24] and 5′-methylthioadenosine (MTA) [25] have been associated with oxidative stress and inflammation, indicating a broader role for ApoJ in hepatic amino acid metabolism. Dysregulation of purine metabolism is linked to insulin resistance, as elevated serum uric acid and related intermediates correlate with impaired insulin secretion [26,27]. The PPP, meanwhile, plays a dual role in diabetes: overactivation promotes oxidative stress and inflammation through NADPH oxidases, while insufficient flux impairs β-cell NADPH production, compromising glutathione recycling and insulin secretion [28]. Therefore, the observed downregulation of these pathways in HKO mice likely reflects reduced oxidative stress and improved insulin sensitivity, consistent with observed metabolic improvements.

Bile acids also emerged as potential mediators of ApoJ-dependent metabolic regulation. Tauroursodeoxycholic acid (TUDCA) has been shown to reduce endoplasmic reticulum (ER) stress, enhance beta-cell function, and improve insulin sensitivity [29,30]. Altered levels of TUDCA-related metabolites in the HKO livers suggest that ApoJ may modulate bile acid signaling pathways, essential for glucose homeostasis and liver function in T2DM.

Moreover, several metabolites derived from dietary sources or the gut microbiota, including betaine, melibiose, and violacein, were also affected by ApoJ deletion. Betaine supplementation has been reported to enhance insulin sensitivity and reduce hepatic lipid accumulation via fibroblast growth factor (FGF) 21-dependent mechanisms [31,32]. Changes in melibiose-derived advanced glycation end-products (MAGEs) and microbial metabolites such as violacein indicate that ApoJ may also influence the gut–liver metabolic axis, thereby affecting systemic glucose and lipid homeostasis [33,34]. Vitamin-related metabolites, including thiamine (B1) and pyridoxal phosphate (B6), were also altered, aligning with previous reports linking deficiencies in these micronu-trients to diabetic complications [35,36].

Despite these findings, several limitations should be acknowledged. Although our data highlight the role of ApoJ in maintaining hepatic metabolic homeostasis under diabetic conditions, the precise molecular mechanisms remain unclear. Given that ApoJ exerts chaperone activity, it may stabilize downstream client proteins during nutrient overload. Indeed, our previous work demonstrated that ApoJ prevents mTOR ubiquitination, thereby inhibiting autophagy [6]. Future studies, employing ApoJ interactome analyses could identify specific client proteins and elucidate the molecular mechanisms underlying its metabolic effects. Furthermore, we did not investigate T2DM-related complications in HKO mice. As HKO markedly improved hepatic metabolism, it is plausible that it may also ameliorate systemic complications such as dyslipidemia, hyperglycemia, and tissue injury. These possibilities warrant future investigation.

## 5. Conclusions

In summary, our findings indicate that hepatocyte-specific ApoJ deletion reshapes hepatic metabolic networks across lipid-, amino acid-, bile acid-, and microbiota-associated pathways. These alterations are particularly relevant to T2DM, in which insulin resistance and hepatic metabolic dysregulation drive the development of MASLD. By modulating these key metabolic pathways, ApoJ acts as a central regulator linking hepatic glucose and lipid homeostasis. Collectively, our findings identify ApoJ as a potential therapeutic node that integrates the pathophysiology of T2DM with the onset and progression of MASLD.

## Data Availability

The metabolic datasets supporting the findings of this study are provided in the Supplementary Tables S1 and S2.

## Supplementary Materials

The following supporting information can be downloaded at: https://www.mdpi.com/article/10.3390/metabo15120761/s1, Figure S1: PCA of metabolites; Table S1: Positive Charge; Table S2: Negative Charge.
