# Supplementary material for: Hepatocyte-Specific ApoJ Knockout Improves Metabolic Profiles in the Liver of Diabetic Mice
Source: Metabolites. 2025 Nov 25;15(12):761. doi: 10.3390/metabo15120761 (PMC12734926; doi:10.3390/metabo15120761)
Supplement: Supplementary file 1 [file metabolites-15-00761-s001.zip › 2025_1108_Supplementary figure.pdf]

A

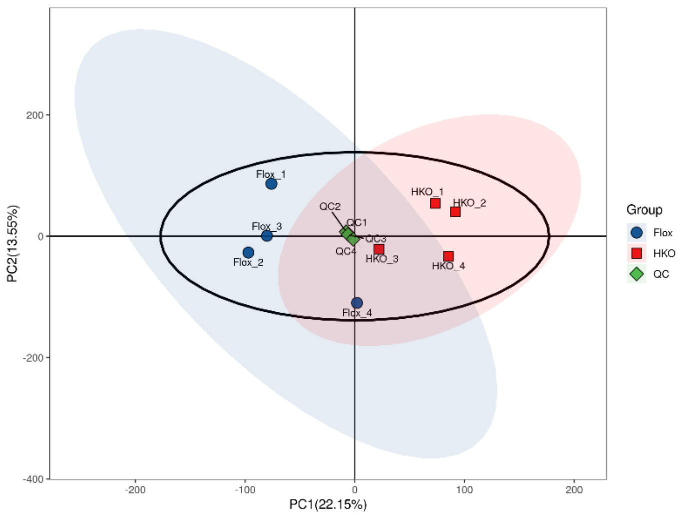

B

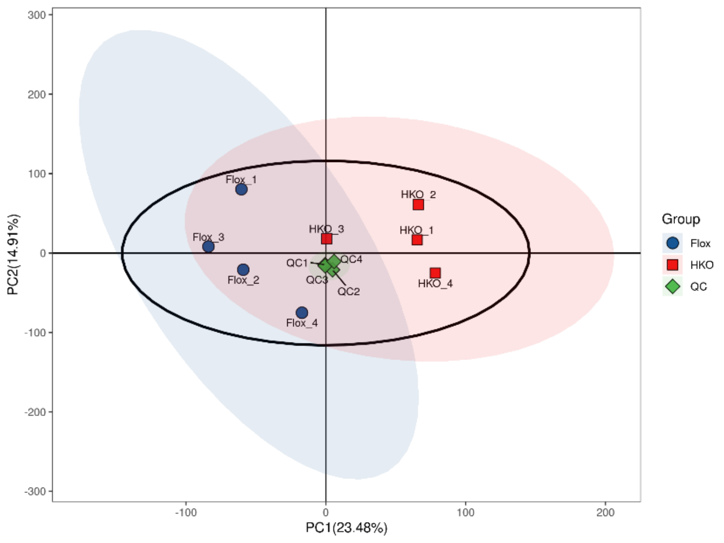

Supplementary Figure S1. PCA analysis of metabolites with positive loadings (A) and negative loadings (B). QC, quality control.
